# Comparison of three exercise interventions with and without gemcitabine treatment on pancreatic tumor growth in mice: No impact on tumor infiltrating lymphocytes

**DOI:** 10.3389/fphys.2022.1039988

**Published:** 2022-11-21

**Authors:** Priti Gupta, Charles F. Hodgman, Claudia Alvarez-Florez, Keri L. Schadler, Melissa M. Markofski, Daniel P. O’Connor, Emily C. LaVoy

**Affiliations:** ^1^ Department of Health and Human Performance, University of Houston, Houston, TX, United States; ^2^ Sheikh Khalifa Bin Zayed Al Nahyan Institute for Personalized Cancer Therapy, MD Anderson Cancer Center, Houston, TX, United States; ^3^ Department of Pediatrics-Research, Division of Pediatrics, The University of Texas MD Anderson Cancer Center, Houston, TX, United States

**Keywords:** chemotherapy, pancreatic ductal adenocarcinoma, physical activity, tumor infiltrating lymphocytes, natural killer cells

## Abstract

Exercise has been shown to slow pancreatic tumor growth, but whether exercise interventions of differing volume or intensity yield differential effects on tumor outcomes is unknown. In this study, we compared three exercise training interventions implemented with and without chemotherapy on pancreatic tumor growth in mice.

**Methods:** Male C57BL/6 mice (6–8 weeks old) were subcutaneously inoculated with pancreatic ductal adenocarcinoma tumor cells (PDAC 4662). Upon tumor detection, mice received gemcitabine 15 mg/kg intraperitoneally 3 days/week and were assigned to exercise: high volume continuous exercise (HVCE), low volume continuous exercise (LVCE), high intensity interval training (HIIT), or sedentary (SED). HVCE ran at 12 m/min for 45 min and LVCE for 15 min, 5 days/week. HIIT ran 1-min at 20 m/min, followed by 1-min walking at 8 m/min for 20 total intervals, 3 days/week. SED did not run. Additional sets of inoculated mice were assigned to the exercise interventions but did not receive gemcitabine. Tumor volume was measured every other day for 2 weeks; tumor-infiltrating lymphocytes were assessed by flow cytometry 3-week post-inoculation.

**Results:** Tumor growth did not differ between groups that received gemcitabine (F_(3, 34)_ = 1.487; *p* = 0.235; η^2^ = 0.116). In contrast, tumor growth differed between groups not provided gemcitabine (F_(3,14)_ = 3.364; *p* = 0.049, η^2^ = 0.419), with trends for slower growth in LVCE than SED (*p* = 0.088) and HIIT (*p* = 0.084). Groups did not differ in tumor infiltrating lymphocytes.

**Conclusion:** Contrary to our hypotheses, the exercise interventions compared here did not further reduce pancreatic tumor growth beyond that provided by gemcitabine. However, in mice not receiving gemcitabine, there was a trend for reduced tumor growth in LVCE.

## 1 Introduction

Pancreatic cancer is a highly lethal disease with only a 9% 5-year survival rate (Siegel et al., 2019). It is the fourth leading cause of cancer-related death in the United States (Siegel et al., 2019). A small number of studies have investigated whether physical exercise can benefit patients with pancreatic cancer. Both cardiorespiratory and resistance exercise appears to be tolerated and can reduce psychological distress and improve physical function during and after treatment (Yeo et al., 2012; Ngo-Huang et al., 2017; Ausania et al., 2019; Wiskemann et al., 2019). Despite these benefits, many oncologists still fail to recommend exercise, due in part to concerns of its tolerability as well as lack of knowledge of a precise exercise prescription (Park et al., 2015; Smaradottir et al., 2017).

High volume (225 min a week) moderate intensity exercise in combination with chemotherapy has been shown to reduce pancreatic tumor growth in mice (Schadler et al., 2016). Whether a smaller volume of moderate intensity exercise can also yield benefit is unknown. The effects of high intensity exercise on pancreatic cancer outcomes are also unknown but could be expected to yield different results than moderate intensity exercise. Exercise at different intensities confers different physiologic and gene expression adaptations in healthy tissues as well as in different diseases, including cancer (Wisløff et al., 2007; Sasso et al., 2015; Stout et al., 2017). In rat models of mammary carcinogenesis, the degree of protection against tumor growth increases with exercise intensity (Thompson et al., 1995; Malicka et al., 2015). High intensity interval training (HIIT) has been shown to reduce lung tumor metastases and tumor volume in mouse models of breast cancer, and may increase levels of antitumor cytokines in circulation (Barra et al., 2017; Devin et al., 2019; Nezamdoost et al., 2020). Whether pancreatic tumor outcomes are also differentially impacted by different exercise intensities remains to be determined.

Animal models investigating the direct effect of exercise on pancreatic tumor growth have frequently reported the effect of exercise offered alone, without additional cancer treatments (Zheng et al., 2008; Song et al., 2017; Kurz et al., 2022). Results from these studies may be difficult to translate to clinical practice, as in clinical settings exercise would almost certainly be provided as an adjuvant therapy to, not *in lieu* of, surgery, radiation, chemotherapy, or immunotherapies. While exercise training has shown benefit in reducing cardiotoxic effects of certain chemotherapies (Gomes-Santos et al., 2021), the impact of exercise and chemotherapy together on tumor-specific outcomes has been less frequently considered. Thus, a more complete understanding of the effects of various exercise interventions in conjunction with other treatments during pancreatic cancer is required.

Cytotoxic lymphocytes, including CD8^+^ T-cells and Natural Killer (NK) cells, are integral in the immune response against cancer. CD8^+^ T-cells can identify and kill malignant cells, and NK cells can eliminate MHC-I deficient tumor cells that have escaped CD8^+^ T-cells (Dunn et al., 2004). Exercise is known to affect immune cell function in an intensity- and duration-dependent manner, and enhanced immunosurveillance and activity has been suggested as a mechanism underlying the beneficial effects of exercise on cancer risk and progression (Walsh et al., 2011; Christensen et al., 2018). Both CD8^+^ T-cells and NK cells are transiently mobilized into peripheral blood by exercise, increasing in concentration in a manner proportional to exercise intensity and duration (Shephard and Shek, 1999; Walsh et al., 2011; LaVoy et al., 2017). NK cell cytotoxicity against tumor cells is increased after exercise (Shephard and Shek, 1999; Bigley et al., 2014). Exercise may also enhance lymphocyte infiltration into tumors, and increased tumor infiltrating lymphocytes (TIL) have been associated with decreased tumor growth in exercised mice (Pedersen et al., 2016; Song et al., 2017; Kurz et al., 2022). Whether different exercise training protocols will yield differences in TIL has not been determined.

In the current study, we first aimed to shed light on whether differences in exercise training volume and intensity would yield differences in pancreatic tumor growth and the proportion of TIL when exercise was included as an adjuvant to chemotherapy. Interventions were initiated after tumors were detected and were implemented alongside gemcitabine (GEM). Gemcitabine is a chemotherapy drug that induces DNA damage and has been a standard treatment for patients with pancreatic cancer (Heinemann, 2001; Nishimoto, 2022). As the existing literature has primarily focused on the effects of exercise training without chemotherapy, and as chemotherapy could mask differences between exercise protocols, we separately aimed to compare three different exercise interventions on pancreatic tumor growth in mice without chemotherapy treatment. As greater exercise intensities have been shown to yield greater reductions in tumor growth, and enhanced immune cell mobilization, we hypothesized that exercise training would slow tumor growth in an intensity and duration-dependent manner, and that this effect would be related to increased TIL. Specifically, we hypothesized that high intensity interval training would yield slower tumor growth and increased TIL compared to moderate intensity exercise, and that longer duration moderate intensity exercise would yield slower tumor growth, increased TIL, and enhanced NK cell function compared to shorter duration moderate intensity exercise. We hypothesized that these effects would be observed with and without gemcitabine.

## 2 Materials and methods

### 2.1 Cell culture

Pancreatic ductal adenocarcinoma (PDAC-4662) tumor cells were originally generated from Kras^LSL−G12D/-,^ Trp53^LSL−R172H/+,^ Pdx1-Cre (KPC) mice and were provided by Dr. Robert Vonderheide (University of Pennsylvania School of Medicine). Cells were preserved in a protein-free, sterile cryopreservation medium containing 10% DMSO in cryovials (USA Scientific, Ocala, FL) in liquid nitrogen. A week before inoculation, cell lines were thawed and maintained in Dulbecco’s Modified Eagle Medium (Genesis Scientific, San Diego, CA) containing 2 mm glutamine, 10% Fetal Bovine Serum, 100 U/mL penicillin and 100 μg/ml streptomycin. We have previously shown these cells to be highly desmoplastic (Schadler et al., 2016).

### 2.2 Animal experiments and exercise interventions

All animal experiments were approved by the Institutional Animal Care and Use Committee at MD Anderson Cancer Center, Houston, Texas (protocol number 00001380-RN02). Six-week-old male C57BL/6 mice were purchased from Jackson Laboratories. Mice were maintained in standard housing cages in a thermo-stated environment under a 12 h light/dark cycle with access to food (normal chow, 2,844 kcal/kg, 4% crude fat) and drinking water *ad libitum*. Mice were acclimatized to the treadmill (Columbus Instruments) over 3 days by brief (5 min) exposure to walking at 8 m/min. Following acclimatization, 3 × 10^5^ tumor cells in 200 µL PBS were injected subcutaneously into the shaved flanks of mice. Prior work in our laboratory had established that this number of cells led to consistent tumor formation that developed over the course of 3 weeks. As expected, the tumors that developed were very dense and had defined capsules visually apparent as a thick, glossy layer surrounding each tumor. These subcutaneous tumors were not expected to metastasize (Miquel et al., 2021).

#### 2.2.1 Experiments with gemcitabine

When tumor volume reached ≥50 mm^3^ (on average, 55 mm^3^,7–8 days post-inoculation), mice were assigned to one of four experimental groups (*n* = 6/group): no exercise and gemcitabine (SED + GEM); Low Volume Continuous Exercise and gemcitabine (LVCE + GEM); High Volume Continuous Exercise and gemcitabine (HVCE + GEM); or High Intensity Interval Training and gemcitabine (HIIT + GEM). Group assignments were made to ensure that groups were approximately equal regarding tumor volume at the start of the intervention. Gemcitabine (15 mg/kg) was administered intraperitoneally 3 days/week. This dosage of gemcitabine was selected as prior work in our laboratory established that it slows but does not completely abrogate tumor growth. LVCE + GEM and HVCE + GEM ran on a treadmill at a speed of 12 m/min for five consecutive days/week, for 15 and 45 min, respectively. The exercise protocol for HVCE was selected as it has previously been shown to be tolerated in pancreatic tumor-bearing mice receiving GEM, and to reduce pancreatic tumor growth (Schadler et al., 2016). While we did not conduct maximal exercise tests in the present study, this speed approximates 65–75% VO_2max_ in adult C57BL/6 mice (Schefer and Talan, 1996; Mille-Hamard et al., 2012). LVCE was selected to determine whether benefits could be derived from exercise of the same intensity but of 1/3 the volume. Animals in the high intensity interval training group (HIIT + GEM) performed 10 intervals of 1 min running at 20 m/min followed by 1 min walking at 8 m/min, three non-consecutive days/week. This speed approximates 90–100% VO_2max_ (Schefer and Talan, 1996; Mille-Hamard et al., 2012). Similar protocols have been shown to be feasible in tumor-bearing C57/BL/6 mice (Barra et al., 2017; Zhang et al., 2020). Exercise sessions were performed at the same time each day. All mice were encouraged to run by tapping the animals’ backs with a bottle brush if they drifted towards the end of the treadmill. Sedentary (SED + GEM) mice did not run but were transferred to an empty cage, without food and water access for 15 min, 5 days/week.

Tumor size was measured using calipers every other day and tumor volume was calculated as [(length*length*width)/2]. Tumor measurements were performed by the same individuals administering the intervention and thus evaluators were not blinded to group assignment. Mice were euthanized 3-weeks after tumor inoculation, 24–48 h following the last exercise session and/or GEM treatment. This experiment was replicated as described above but using an additional 25 mice (*n* = 5/group). Tumor growth within treatment groups did not differ between the two replications (all *p* >0 .05 in comparison, data not shown), and so results from both experiments were combined in the analyses.

In three mice (one each in SED + GEM, HVCE + GEM and HIIT + GEM) tumor growth did not continue after group assignment; these mice were not included in final analyses. An additional animal was not included in final analyses as it refused to run on any session (HIIT + GEM).

#### 2.2.2 Experiments without gemcitabine

**FIGURE 1:**
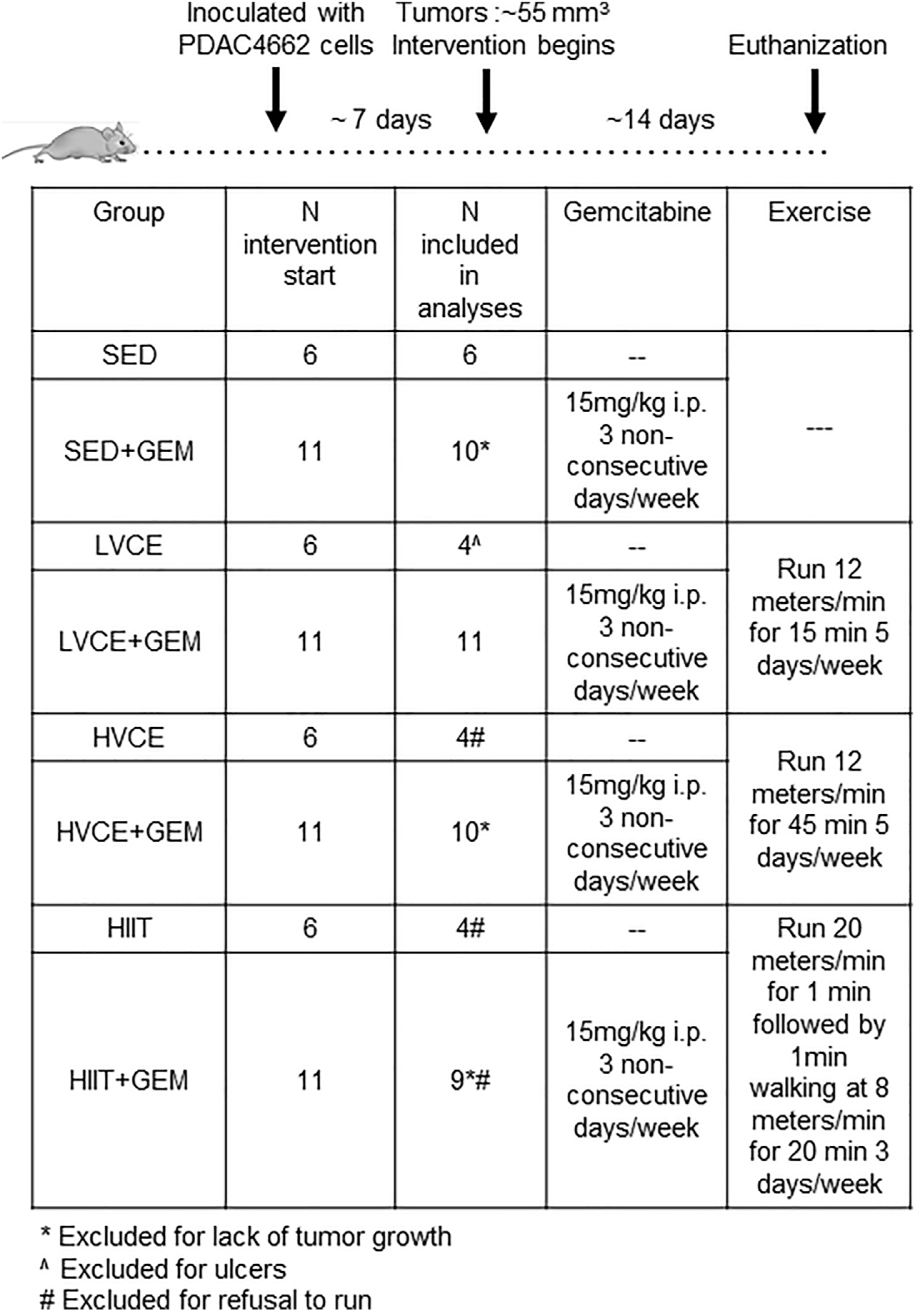
Experimental design. SED: Sedentary; GEM: gemcitabine; LVCE: low-volume continuous exercise; HVCE: high-volume continuous exercise; HIIT: high intensity interval training.

A similar experiment as described above was conducted with an additional 24 animals; the experiment only differed in that mice were not provided gemcitabine. When tumors reached approximately 55 mm^3^ (8 days post-inoculation), mice were assigned to one of four groups (*n* = 6/group): no exercise (SED); Low Volume Continuous Exercise (LVCE); High Volume Continuous Exercise (HVCE); or High Intensity Interval Training (HIIT). Tumors were measured every other day using calipers. Mice were euthanized 3-weeks after tumor inoculation, except two mice in LVCE that were euthanized at day 17 due to ulcer development; these mice were excluded from analyses. An additional three animals were not included in final analyses as they refused to run on any session (HVCE: *n* = 2, HIIT: *n* = 1), and one animal in HIIT was excluded for completing less than 50% of sessions. The experimental design is illustrated in Figure 1.

### 2.3 Immune assays

At the end of intervention, mice were euthanized, and blood, spleen, and tumor were collected. Tumor infiltrating immune cells were isolated from tumor by incubating minced tumor sections in a collagenase mixture (40 mg Worthington Type I Collagenase +0.3 mg Worthington DNAase in 20 ml HBBS solution (Thermo Fisher Scientific, Waltham, MA) for 35 min followed by passage through 100 μm and 70 µm strainers. The filtrate was washed, resuspended in PBS and stained with fluorescent monoclonal antibodies (Miltenyi Biotech Inc., San Diego, CA) specific for cell-surface markers: CD3-PE-Vio770, CD4-Viogreen/Vioblue, Nkp46-FITC, and CD8a-APC Vio770. Blood was collected via cardiac puncture into EDTA coated tubes. Blood was treated with red blood cell lysis buffer (eBiosciences Inc., San Diego, CA) and stained with fluorescent monoclonal antibodies as above. Labeled cells were analyzed with a MACSQuant analyzer flow cytometer (Miltenyi Biotec Inc.). Compensation beads (Miltenyi Biotech Inc.) were used to compensate for spectral overlap in each panel. Florescence minus one (FMO) controls were used for gating. Single color tubes were used to further identify positive and negative staining by each antibody.

### 2.4 Statistical analysis

Prior research has demonstrated a medium-to-large effect of exercise on tumor growth in PDAC4662-inoculated mice treated with gemcitabine (Schadler et al., 2016). Thus, *n* = 6 per group was expected to provide 80% power to detect differences in tumor growth due to exercise at *p* < 0.05. Additional mice were used in these experiments as we were not aware *a priori* of the effect size for differences between exercise protocols in this model. Data were screened for outliers and normality by visual inspection of histograms and Q-Q plots and by examining skewness and kurtosis statistics; data was transformed when needed. Tumor growth (that is, tumor volume measured across intervention period) within each group of mice in the first set of experiments with GEM was compared with tumor growth in the corresponding group of mice in the second set of experiments with GEM; as groups did not differ in tumor growth the groups were combined in subsequent analyses. Mixed effects repeated measures Analysis of Variance (ANOVA) with Greenhouse-Geisser correction were used to examine the effects of each exercise intervention on body weight and tumor growth over time. One-way ANOVA were used to compare body weight, total distance run, initial and final tumor volume and final tumor volume/final body weight between each group on the final measurement day, as well as to assess group differences in immune data. Data were further explored by pairwise comparisons with adjustments for multiple comparisons by the method of Sidak. All statistical analyses were performed using “Statistical Package for the Social Sciences” version 21 software (IBM Corp, 2019). Statistical significance was accepted at *p* < 0.05. Results presented in figures contain non-transformed data.

## 3 Results

### 3.1 Exercise training completion and body weight

The majority of animals in each exercise group were able to complete their assigned intervention without protocol modification. One mouse in LVCE + GEM refused to run on one session and one mouse in HIIT + GEM ran nine of 10 intervals on the final session; these animals were included in the analyses. One mouse in HIIT + GEM refused to run on any session and so completed 0% of their assigned intervention; these mice were not included in final analyses. Two mice in LVCE required early euthanization due to ulcer development and thus completed 70% of planned exercise sessions; these animals were not included in the analyses. Two mice in HVCE and two mice in HIIT refused to run on any session and so completed 0% of their assigned intervention; these mice were not included in final analyses. All other mice were able to complete all assigned exercise sessions. Over the course of the intervention, animals in HVCE and HVCE + GEM ran on average significantly farther than other groups; LVCE, LVCE + GEM, HIIT, and HIIT + GEM did not differ in total distance run (Table 1).

**TABLE 1:** Distance run and body weight at beginning and end of intervention. Data reported are mean ± SD; *p*-value for group differences.

	SED	SED + GEM	LVCE	LVCE + GEM	HVCE	HVCE + GEM	HIIT	HIIT + GEM	p-value
Total distance run (m)	-	-	1800.00 ± 0.00^a^ ^,^ ^b^	1783.63 ± 54.27^a^ ^,^ ^b^	5400.00 ± 0.00	5400.00 ± 0.00	1680.00 ± 0.00^a^ ^,^ ^b^	1676.89 ± 9.33^a^ ^,^ ^b^	<0.001
Body weight (g) at start	24.81 ± 1.68	23.71 ± 2.20	23.49 ± 0.68	23.00 ± 2.15	21.88 ± 1.12	22.78 ± 1.81	23.26 ± 1.38	23.15 ± 2.02	0.381
Body weight (g) at end	26.00 ± 1.97	25.14 ± 2.06	24.63 ± 0.39	23.69 ± 2.52	23.81 ± 21.88	23.11 ± 1.46	25.09 ± 1.53	24.05 ± 2.01	0.169

^a^ differs from HVCE,

^b^ differs from HVCE+GEM.

Groups did not differ at the beginning or end of the intervention in body weight (Table 1). Body weight significantly increased over time in all animals (F_(3.9,188)_ = 45.258, *p* ≤ 0.001, η^2^ = 0.485). Groups did not differ in body weight gain (F_(7,48)_ = 1.248; *p* = 0.296, η^2^ = 0.154).

### 3.2 Tumor growth

**FIGURE 2:**
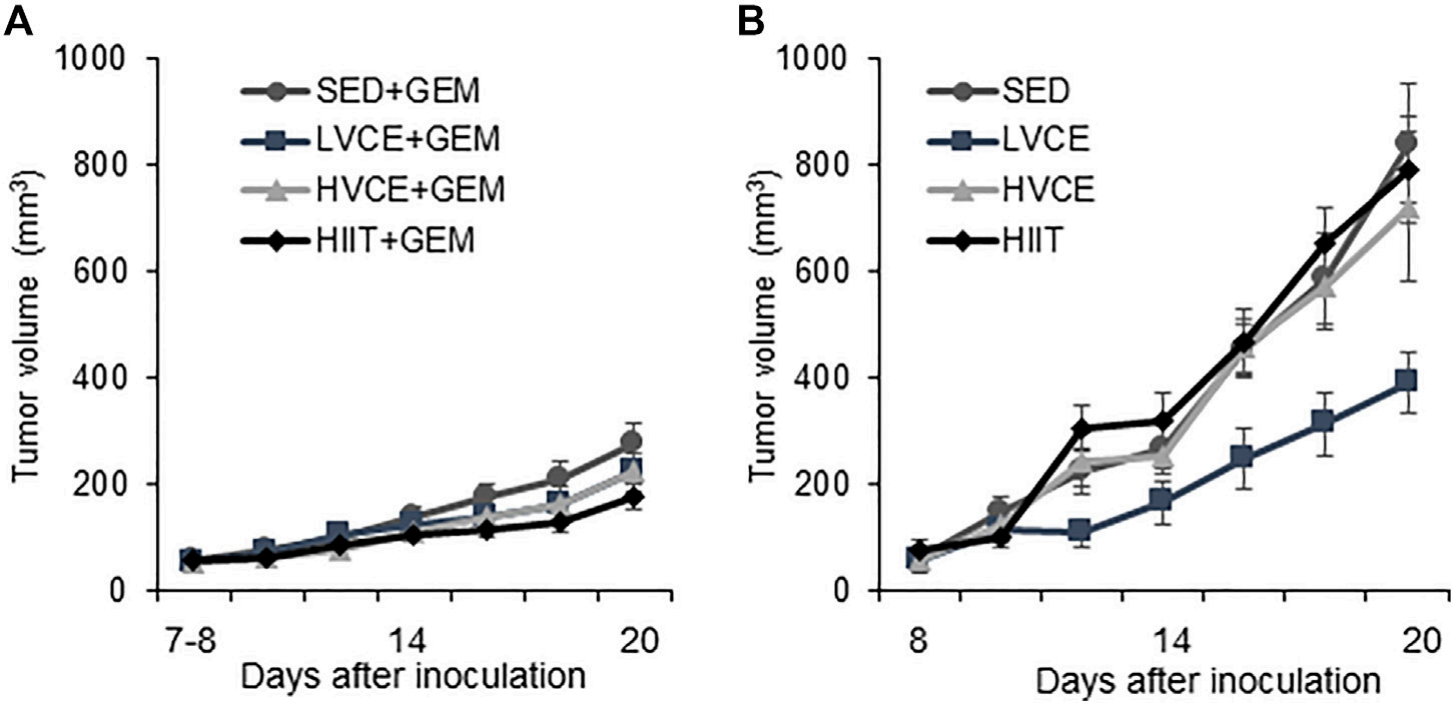
Tumor growth in sedentary and exercised mice. **(A)** Tumor volume over the course of the intervention in mice treated with gemcitabine, *n* = 10 (SED + GEM), *n* = 11 (LVCE + GEM), *n* = 10 (HVCE + GEM), *n* = 9 (HIIT + GEM). **(B)** Tumor volume over the course of the intervention in mice without gemcitabine, *n* = 6 (SED), *n* = 4 (LVCE), *n* = 4 (HVCE), *n* = 4 (HIIT). Tumor volume shown as mean ± SEM.

Tumor volume did not differ between any group at the start of the interventions (F_(7,55)_ = 0.353; *p* = 0.925, η^2^ = 0.049). As shown in Figure 2A, tumor volume significantly increased over time in all animals receiving gemcitabine (F_(2.4,84)_ = 80.533; *p* = 0.001, η^2^ = 0.703). SED + GEM, LVCE + GEM, HVCE + GEM, or HIIT + GEM did not differ in tumor growth (F_(3, 34)_ = 1.487; *p* = 0.235; η^2^ = 0.116).No overall differences between groups in tumor volume were detected on the final tumor measurement day (F_(3,34)_ = 1.795; *p* = 0.167, η^2^ = 0.137). Groups did not differ in final tumor volume relative to final body weight (mm^3^/g) (F_(3,34)_ = 1.544; *p* = 0.221, η^2^ = 0.120).

Tumor volume also significantly increased over time in all animals not receiving GEM (F_(2.3, 31.7)_ = 51.546; *p* ≤ 0.001, η^2^ = 0.786) (Figure 2B). Tumor growth significantly differed between SED, LVCE, HVCE, and HIIT (F_(3,14)_ = 3.364; *p* = 0.049, η^2^ = 0.419). Although not significant, post-hoc analyses reveal a trend for lower tumor volume in LVCE compared to SED (LVCE: mean average tumor volume ±SE: 200.69 ± 38.75 mm^3^, SED: 370.56. ± 45.68 mm^3^; *p* = 0.088) and HIIT (388.53 ± 45.77 mm^3^; *p* = 0.084), but not compared to HVCE (348.02 ± 42.28 mm^3^, *p* = 0.248). Groups did not differ in tumor volume at the end of the experiment (F_(3, 14)_ = 2.915; *p* = 0.071, η^2^ = 0.384), nor did groups differ in final tumor volume relative to final body weight (F_(3,14)_ = 2.773; *p* = 0.080, η^2^ = 0.373).

### 3.3 Lymphocyte responses

**FIGURE 3:**
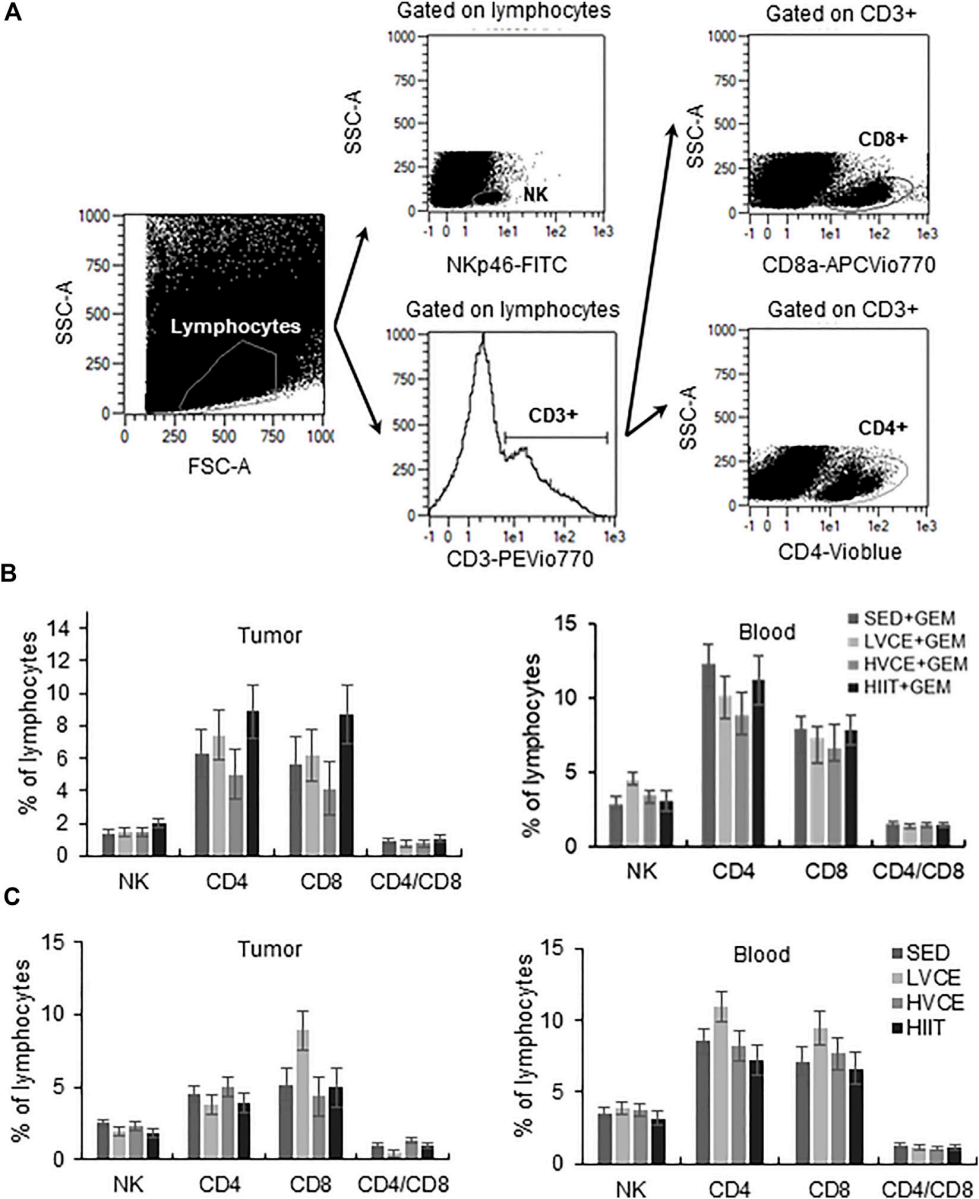
Tumor infiltrating and peripheral blood lymphocytes at the end of the interventions. **(A)** Representative flow cytometry plots illustrating gating strategy to identify TIL. A similar gating strategy was followed for blood samples. **(B)** Lymphocytes isolated from tumor (left plot) and blood (right plot) in GEM animals; *n* = 6 (SED + GEM), *n* = 6 (LVCE + GEM), *n* = 6 (HVCE + GEM), *n* = 5 (HIIT + GEM). **(C)** Lymphocytes isolated from tumor (left plot) and blood (right plot) in animals without GEM; *n* = 6 (SED), *n* = 4 (LVCE), *n* = 4 (HVCE), *n* = 4 (HIIT). Data shown as mean ± SEM.

Exercise did not alter the proportions of lymphocytes found in tumors from mice treated with GEM (Figure 3B). Likewise, peripheral blood lymphocytes did not significantly differ between groups (Figure 3B). In mice not treated with GEM, groups did not differ in the infiltration of lymphocytes into tumors, nor did peripheral blood lymphocytes differ between groups (Figure 3C).

## 4 Discussion

We compared the effects of three different exercise training interventions offered with and without GEM on tumor growth in a mouse model of pancreatic cancer. We report no significant difference in tumor growth due to exercise in animals that received GEM. These results suggest that exercise initiated after tumor development may not add tumor-specific benefit beyond that provided by GEM in this subcutaneous PDAC4662 model of pancreatic cancer. However, groups that did not receive GEM differed in tumor growth. Although not significant, there was a trend for reduced tumor growth in LVCE.

We hypothesized that exercise of greater intensity and longer duration would yield slower tumor growth. This hypothesis was rooted in the existing literature demonstrating exercise training reduces tumor growth in an intensity- and duration-dependent manner across several cancer models (Thompson et al., 1995; Singh et al., 2005; Malicka et al., 2015). However, while not significant, the group that performed the least exercise exhibited the smallest tumors. Although we urge caution in interpreting this nonsignificant result, a low volume of moderate intensity exercise has been shown to reduce tumor growth in a mouse model of liver cancer (Zhang et al., 2016). This line of research investigating exercise of low volume and intensity is important, as it may be easier for cancer patients to complete smaller bouts of exercise. However, the majority of the mice in the present study that began their exercise interventions were able to complete their assigned protocols without modification, suggesting each protocol was well-tolerated. In the current study, there was no difference between HIIT and SED. HIIT has been shown to reduce tumor burden in a mouse model of breast cancer (Barra et al., 2017). Indeed, exercise intensity seems to be important in breast cancer, as the reduction in mammary tumors tends to directly relate to exercise intensity (Malicka et al., 2015). Conversely, continuous exercise has been shown to be superior to HIIT at reducing liver tumor incidence, suggesting that the effects of different exercise interventions differ between murine cancer types (Zhang et al., 2020). We are not aware of another study that has used HIIT in mouse models of pancreatic cancer.

While the studies mentioned above examined the effects of exercise alone, others have demonstrated that exercise training may add additional anti-tumor benefit beyond chemotherapy alone (Khori et al., 2015; Schadler et al., 2016; Wennerberg et al., 2020). The current study aimed to add to this literature by for the first time comparing different exercise interventions provided with GEM. One of our exercise interventions, HVCE + GEM, was modeled from an exercise intervention previously shown to significantly reduce PDAC4662 pancreatic tumor growth when provided with GEM relative to GEM alone (Schadler et al., 2016). However, we found no differences in tumor growth between our exercise and sedentary groups. Some methodological differences could have contributed to the discrepancy between our results and those reported previously. First, the route of chemotherapy delivery differed between the studies, which could have led to differences in bioavailability (Pestieau et al., 1998). A second possible explanation is a difference in the timing of the intervention relative to tumor growth. Schadler and colleagues initiated exercise earlier in tumor development than the current study (i.e. tumor volume of ∼35 mm^3^ compared to ∼55 mm^3^) (Schadler et al., 2016). Beginning the intervention at the larger tumor volume as in the current study may better reflect when patients are diagnosed, as 87% of PDAC patients are diagnosed beyond stage 1 (ASCO, 2022). However, exercise may have a greater effect when initiated at the early stage of the disease, and these beneficial effects are mitigated as the disease progresses. This effect has most frequently been demonstrated when examining exercise as a monotherapy. For example, exercise training was shown to slow the progression of fast-growing B16 melanoma tumors when begun prior to tumor cell inoculation but had no effect on tumor growth when begun after tumor cell inoculation (Pedersen et al., 2016). Similar results have also been reported with mammary tumors, melanoma, and with colon cancer (Shalamzari et al., 2014; Kelly et al., 2017; Buss et al., 2020). It should be noted that other differences exist between these studies and the current, including animal sex, tumor model, and training protocol, thus complicating interpretation. More research is needed to understand the effect of the timing of exercise initiation relative to tumor growth when offered as adjuvant therapy.

We examined tumor infiltrating lymphocytes as a potential mediator of anti-tumor effects. In accordance with our finding of no difference in tumor growth between exercise groups with chemotherapy, we also found no differences in TILs between SED + GEM, LVCE + GEM, HVCE + GEM, and HIIT + GEM. In contrast, despite differing in tumor growth, groups not receiving GEM did not differ in TIL. Previous studies demonstrated that exercise training increases the infiltration of immune cells in B16F10 melanoma tumors (Pedersen et al., 2016) and in orthotopic pancreatic ductal adenocarcinoma tumors (Kurz et al., 2022), and that exercise-induced increases in TIL are associated with tumor regression (Zielinski et al., 2004; Kurz et al., 2022). In a detailed analysis of the tumor-immune environment, Wennerberg and colleagues report an increase in activated, CD8^+^ T-cells and NK cells and a decrease in immune-suppressing myeloid-derived suppressor cells within tumors in a mouse model of breast cancer following 3 weeks of adjuvant exercise training (Wennerberg et al., 2020). The exercise intensity used by Wennerberg et al. was of a fairly high intensity and moderate volume (30 min for 5 days/week at 18 m/min). Finally, exercise may modulate the tumor microenvironment and thus impact tumor-immune interactions (Buss and Dachs, 2020). Future studies would benefit from the inclusion of immunohistochemistry and pathophysiology explorations of the tumor.

To our knowledge, this is the first study comparing the effects of the three different exercise interventions initiated after tumor development on tumor growth with and without GEM. Strengths of this study include the study design, which examined the adjuvant effect of exercise offered alongside chemotherapy and initiated interventions following tumor detection. We also included groups not administered GEM, allowing some comparison to much of the existing literature examining exercise as a neoadjuvant therapy. Secondly, we approximated exercise volumes and intensities that might be achieved in pancreatic cancer patients (Yeo et al., 2012; Ngo-Huang et al., 2017). For example, it is hard to imagine many patients being able to perform daily runs lasting 3 h (Zielinski et al., 2004). Finally, this study included experiments designed to investigate potential mechanisms underlying the effects of exercise on tumor growth, as we measured lymphocytes in tumors and blood.

Our study is not without limitations. First, we did not measure physiological adaptations to exercise, meaning the cardiorespiratory impact of the different exercise interventions is unknown. Our exercise intervention was also quite short. As the goal was to examine the effect of exercise when initiated after tumor detection and this tumor model develops very rapidly, a longer intervention was not possible. However, we (Schadler et al.,2016) and others (Kurz et al., 2022) have demonstrated an effect of just 2 weeks of exercise on pancreatic tumor growth. Second, it is possible that our exercise interventions induced a stress response. Using treadmills rather than voluntary running wheels allowed the comparison of exercise intensity and duration. However, treadmill running may induce a stress response in mice, with increased levels of catecholamines and glucocorticoids that may negatively impact the immune system and tumor outcomes (Simon et al., 1984; Moreno-Smith et al., 2010; Svensson et al., 2016). We did not measure catecholamines or glucocorticoids in the current study, which may be a useful consideration for future studies. It also must be acknowledged that it is difficult to translate exercise interventions from mice to humans. An additional source of stress for all animals in the study is that the mice were run during the period of the day with light, meaning that the mice were awakened from their sleep cycle/lower activity period to run. In the future, we recommend running mice as close to their natural awake cycle as possible. An additional limitation includes the fact that the individuals who measured tumor volume were not blinded to group allocation. We report here the effect of the interventions on tumor volume, but not tumor weight. These data could have provided additional insight into the effects of the intervention. A further limitation is the relatively young age (6–8 weeks) of mice in this study. While this age group aligns with much of the existing literature, almost 90% of pancreatic cancer is diagnosed after the age of 55 (Ilic and Ilic, 2016). We also chose to only use male mice in this study, due to the potential influence of sex hormones on outcomes (Nipp et al., 2018; Zhong et al., 2022). Thus, future studies with older animals and both male and female animals would improve research translation (Dutta and Sengupta, 2016). Finally, we selected a subcutaneous tumor model for its ease of measurement of growth. Different results may have been obtained with an orthotopic model, which is reported to better mimic the tumor microenvironment, disease progression, and metastasis of human disease (Erstad et al., 2018). Given differences in tumor growth, vascularization, immune infiltration, and sensitivity to anti-tumoral treatments between the models, the effects of exercise training on orthotopic tumors may differ and should be examined in future research.

In conclusion, we report no differences in tumor growth due to exercise when offered as an adjuvant to chemotherapy in our subcutaneous PDAC model. However, significant differences in tumor growth were noted in groups that exercised without chemotherapy. No differences in tumor infiltrating lymphocytes were noted.

## Data Availability

The raw data supporting the conclusions of this article will be made available by the authors, without undue reservation.
